# Tiling array data analysis: a multiscale approach using wavelets

**DOI:** 10.1186/1471-2105-12-57

**Published:** 2011-02-21

**Authors:** Alexander Karpikov, Joel Rozowsky, Mark Gerstein

**Affiliations:** 1 Diagnostic Radiology, Yale University, New Haven, CT, USA; 2 Molecular Biophysics & Biochemistry, Yale University, New Haven, CT, USA

## Abstract

**Background:**

Tiling array data is hard to interpret due to noise. The wavelet transformation is a widely used technique in signal processing for elucidating the true signal from noisy data. Consequently, we attempted to denoise representative tiling array datasets for ChIP-chip experiments using wavelets. In doing this, we used specific wavelet basis functions, *Coiflets*, since their triangular shape closely resembles the expected profiles of true ChIP-chip peaks.

**Results:**

In our wavelet-transformed data, we observed that noise tends to be confined to small scales while the useful signal-of-interest spans multiple large scales. We were also able to show that wavelet coefficients due to non-specific cross-hybridization follow a log-normal distribution, and we used this fact in developing a thresholding procedure. In particular, wavelets allow one to set an unambiguous, absolute threshold, which has been hard to define in ChIP-chip experiments. One can set this threshold by requiring a similar confidence level at different length-scales of the transformed signal. We applied our algorithm to a number of representative ChIP-chip data sets, including those of Pol II and histone modifications, which have a diverse distribution of length-scales of biochemical activity, including some broad peaks.

**Conclusions:**

Finally, we benchmarked our method in comparison to other approaches for scoring ChIP-chip data using spike-ins on the ENCODE Nimblegen tiling array. This comparison demonstrated excellent performance, with wavelets getting the best overall score.

## Background

Tiling arrays have recently become widely used to investigate thousands of biochemical reactions in a parallel fashion. In oligonucleotide microarrays the probes are designed to match parts of the known genomic sequence. Genome tiling arrays include overlapping oligonucleotides designed to cover an entire genomic region of interest. These arrays are able to simultaneously monitor the expression of thousands of genes [1] as well as to identify the transcription factor binding sites [2]. Transcription factors are regulatory proteins which bind to DNA and control the gene transcription or biochemical activity of other regulatory proteins. The experimental technique to identify these regions of activity of the regulatory proteins on DNA involves the hybridization of immunoprecipitated DNA on a tiling microarray (ChIP-chip experiments)[3]. In this paper we examine data from two ChIP-chip experiments in order to identify regions of activity.

One common feature of all microarray experiments is that the signal of interest due to biochemical reactions is contaminated by noise. This noise can be mainly attributed to non-specific cross-hybridization. In the ideal case the oligonucleotides on the microarray only bind targets with exactly the right complementary sequences. In reality, however, lower affinity binding with other, imperfect sequences (known as mismatches) also occurs. In order to correct for this non-specific binding two samples labeled with red and green fluorescent dyes (Cy3 and Cy5) are hybridized on the same tiling array simultaneously. One sample, labeled with red dye, contains DNA immunoprecipitated with antibodies against the transcription factor of interest. Another sample, labeled with green dye, is derived from non-immunoprecipitated, genomic DNA and is used as a negative control.

Several statistical techniques have been developed for microarray data analysis [4-10]. Experimental data coming from the Pol II and histone modification ChIP-chip data show a broad distribution of the lengths of biochemically-active regions. The signal-of-interest on the tiling microarray exhibits triangle-like peaks of different widths [9]. There is both a scale and intensity separation between the useful signal and the noise in ChIP-chip experiments. Most of the existing ChIP-chip data analysis methods do not explicitly exploit this feature.

Signal decomposition using basis functions (wavelets) that resemble peaks makes this separation more apparent. Numerical implementation of the wavelet transformation is called Discrete Wavelet Transform (DWT). Computational cost of DWT scales linearly with the number of input data points (~O(N)). The same is also true for the computation cost of the moving window averaging. One of the major advantages of the DWT over the moving average window method is that DWT gives explicit representation of the signal at different lengthscales and the possibility to choose the type of wavelets for the DWT closely resembling the shape of the peaks we are trying to identify. One of the existing ChIP-chip data analysis methods [10] uses wavelets only as a denoising tool by thresholding the wavelet decomposition at a fixed level.

The peaks were detected not from the wavelet decomposition, but by applying Laplacian of a Gaussian (LoG) edge detector. We describe a method which is capable of delineating peaks of different sizes from the wavelet decomposition coefficients at different levels, and the range of sizes is determined by the algorithm parameters. Our method does not require any data pre-processing. Statistical analysis of the wavelet coefficients produces the probability density function of the signal intensity due to the non-specific hybridization and makes it possible to conduct an unbiased identification of protein binding site locations. Below we describe how to employ a wavelet algorithm to analyze the experimental data.

## Methods

### Mathematical formalism

In this section we will provide an overview of wavelet decomposition. The term "wavelets" is used to refer to a set of localized basis functions which posses a special structure. Wavelets are defined by two mutually orthogonal functions: scaling function *ϕ *and mother wavelet *ψ*. The rest of the basis functions can be obtained by performing dilation and translation operations with the scaling function and the mother wavelet. The fundamental idea behind wavelet analysis is that one can separate data based upon its scale. A very comprehensive review of wavelets can be found in [11].

In this work we use the discrete wavelet transform (DWT) algorithm proposed by Mallat [12]. This algorithm uses scales and positions based on powers of two (i.e. dyadic scales and positions). Mallat's algorithm takes the discrete input signal A(m) of length 2N and decomposes it into two signals: A(m+1) and D(m+1), each of length N. The results of this process are called approximation coefficients and detail coefficients. For ChIP-chip data, the approximation coefficients represent a relevant signal, whereas the detail coefficients represent noise. The approximation coefficients at scale (*m*+1) can be obtained from the approximation coefficients at the finer resolution scale (m) as follows [11]:

(1) $$A{\left(m+1\right)}_{n}=\frac{1}{\sqrt{2}}\sum_{k}c_{k}A{\left(m\right)}_{2n+k}$$

where *c_k_*, *k *= 1,.., 6 are the decomposition low-pass filter coefficients. For the Coif1 wavelet the numerical values are [13]:

*c*_1 _= -0.0157, *c*_2 _= -0.0727, *c*_3 _= 0.3849, *c*_4 _= 0.8526, *c*_5 _= 0.3379, *c*_6 _= -0.0727.

Similarly the detail coefficients at scale (m+1) can be obtained from the approximation coefficients at scale (m) as follows:

(2) $$D{\left(m+1\right)}_{n}=\frac{1}{\sqrt{2}}\sum_{k}b_{k}A{\left(m\right)}_{2n+k}$$

where *b_k_*, *k *= 1,.., 5 are the decomposition high-pass filter coefficients. For the Coif1 wavelet the numerical values of the coefficients are:

*b*_1 _= 0.0727, *b*_2 _= 0.3379, *b*_3 _= -0.8526, *b*_4 _= 0.3849, *b*_5 _= 0.0727, *b*_6 _= -0.0157.

**Figure 1:**
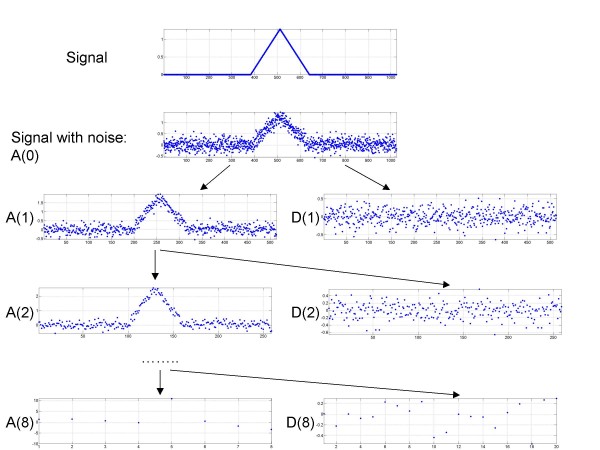
**Schematic representation of the decomposition process**. Raw signal is used as input signal for the approximation coefficients A0.

A schematic view of the decomposition process is shown in Figure 1.

Equations (1) and (2) perform low-pass and high-pass filtering of the input signal. Approximation coefficients retain a low-frequency, smoothed version of the signal; whereas the detail coefficients capture the low-scale, high frequency component of the signal. Procedures (1) and (2) can be performed recursively using A(m+1) as the input signal. In practice, the discrete input signal is treated as the set of approximation coefficients at scale m = 0, and multilevel wavelet decomposition is performed using formulas (1) and (2). The decomposition (1)-(2) is reversible. Approximation coefficients at scale (m) can be reconstructed from the approximation and detail coefficients at scale (m+1):

(3) $$A{\left(m\right)}_{n}=\frac{1}{\sqrt{2}}\sum_{k}f_{n-2k}A{\left(m+1\right)}_{k}+\frac{1}{\sqrt{2}}\sum_{k}g_{n-2k}D{\left(m+1\right)}_{k}$$

For the Coif1 wavelet, the low-pass filter coefficients *f *and high-pass filter coefficients *g *are [13]:

$$\begin{array}{l} f_{1}=-0.0727,f_{2}=0.3379,f_{3}=0.8526,f_{4}=0.3849,f_{5}=-0.0727,f_{6}=-0.0157,\\ g_{1}=-0.0157,g_{2}=0.0727,g_{3}=0.3849,g_{4}=-0.8526,g_{5}=0.3379,g_{6}=0.0727 \end{array}$$

The pyramidal structure of the algorithm makes signal decomposition (1)-(2) and signal reconstruction (3) computationally very efficient [11]. Additional file 1 Figure S1 shows the high-pass and low-pass filter coefficients for the decomposition and reconstruction procedures.

### Specific datasets used for the analysis

All the analysis was performed using data from Nimblegen's ENCODE tiling arrays. The goal of the ENCODE (Encyclopaedia of DNA Elements) project [14] is to identify functional elements from a representative 1% of the human genome. This part of the human genome is represented on the Nimblegen ENCODE tiling array. The single array contains more than 384,000 unique 50-mer probes selected from 30 megabases of human sequence data specified by the ENCODE PROJECT CONSORTIUM [14]. These probes are spaced apart every 38 bases on the average, thus creating a 12-base overlap between probes. No probes were included for interspersed repetitive DNA, thus there are gaps in genome tiling paths on the array. The POL II (CTD4H8) and H3K36ME3 histone modification data were obtained from [15]. It is interesting to use these datasets to test our algorithm because they have broad peaks.

## Results and Discussion

In the current work we propose a new computational approach to analyze ChIP-chip data using wavelet decomposition. A schematic view of the decomposition process is shown in Figure 1.

**Figure 2:**
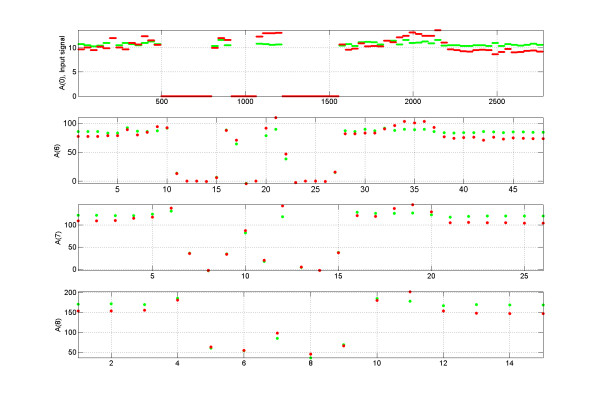
**Input signal and wavelet approximation coefficients of the input signal**. Top graph: An example of the part of the input signal for the wavelet decomposition algorithm. Both red and green channels are shown. The signal is assumed to be zero for the missing data (gaps) in the genomic regions without any probes on the tiling array. Second, third, and fourth graphs from the top: approximation coefficients A(*m*) of the input signal for the decomposition levels *m *= 6, 7, and 8.

We use *Coiflets *(Coif1) as basis functions for the wavelet decomposition [11], as Coiflets have a nearly symmetrical, peak-like form of the mother wavelet. This shape resembles the tiling array signal profile at the transcription factor binding sites observed in ChIP-chip experiments (see top graph in Figure 2). We chose Coiflets for the wavelet decomposition due to their properties of having the maximum number of zero moments while also having small widths (also called support in wavelet literature), ensuring a fast convergence rate [11].

We applied a thresholding procedure to the wavelet coefficients at different resolutions in order to delineate the regions of biochemical activity of interest at the same confidence level for all relevant length-scales.

### Application of wavelet analysis to Pol II and histone modification data

Our goal is to expand both signals in red and green channels using a wavelet basis.

The input signal for the wavelet decomposition is derived as follows. We define the signal as a function of the genomic coordinate to be equal to the measured intensity of this probe for the genomic coordinates inside of the non-overlapping part of the probe, as well as for half of the part which overlaps with the nearest-neighbor probe along the genomic coordinate. Each overlapping region is divided equally between two nearest- probes along the genomic coordinates. The signal is assumed to be zero for the missing data (gaps) in the genomic regions lacking probes on the tiling array. An example of the input signal for the wavelet decomposition algorithm is shown in Figure 2 (top graph). In this figure, the signal of biochemical activity (Pol II binding site in this case) contained in the red channel is located between genomic coordinates 1900 and 2200 and also between 1050 and 1250.

It is important to mention that almost all the wavelet coefficients are greater than one (A(*m*) > 1). As a result it is possible to log transform them. Wavelet coefficients corresponding only to the missing data points (we assigned zero signal to the missing data points) next to the tiled regions can take small values values (A(*m*) < 1) and even can become negative. We filter out those coefficients while retaining the rest, including those corresponding to peaks. Wavelet coefficients A(*m*) > 1 are log-transformed for further analysis.

The level of wavelet decomposition to be used is defined by the typical length-scale of the signal variation we wish to analyze. This length-scale (the size of the peaks) is in the range of 200-2000 base pairs in the ChIP-chip experiments for Pol II data and in the range of 200-4000 base pairs for the histone modification data. The width of the wavelet at the composition level *m *is approximately 2*^m^*. Hence, we used wavelet decomposition levels *m *= 8, 9, 10, 11 for the Pol II data (range: 256-2,048) and *m *= 8, 9, 10, 11, 12 for the histone modification data (range: 256-4,096) to resolve the signal variation length-scales of interest.

### Choice of the wavelet decomposition levels

Approximation coefficients for the wavelet decomposition at levels *m *= 8 through *m *= 12 should capture the signal on the tiling array due to biochemical activity. The presence of this activity is indicated by the enrichment of the red channel signal relative to the green channel signal. If there is no enrichment of the signal in the red channel relative to the signal in the green channel, we expect the wavelet coefficients for the red and green channels to grow proportionally to each other as a function of the average intensity. Wavelet coefficients corresponding to the regions of the enrichment of the red signal relative to the green signal will exhibit deviation from this main trend. The approximation coefficients A(*m*) of the input signal for the decomposition levels *m *= 6, 7, and 8 are shown on the second, third, and fourth graphs from the top in Figure 2. The region of biochemical activity between genomic coordinates 1900 and 2200 is captured by one wavelet approximation coefficient at decomposition level *m *= 8, three wavelet approximation coefficients at decomposition level *m *= 7 and five wavelet approximation coefficients at decomposition level *m *= 6. Broader peaks require higher order wavelet decomposition.

**Figure 3:**
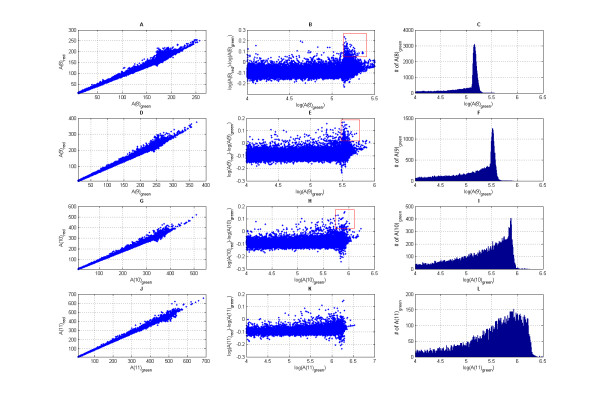
**Wavelet coefficients A(*m*)_red _vs. A(*m*)_green_**. Wavelet coefficients A(*m*)red vs. A(*m*)green for the wavelet decomposition levels *m *= 8, 9, 10, and 11 (subplots A, D, G and J). Each point on the graph corresponds to the pair of the wavelet coefficients of the signals of the red and green channels on the tiling array. The same data is shown in subplots B, E, H, and K using different coordinates: [log(A(*m*)_red_) - log(A(*m*)_green_)] vs. A(*m*)_green_. Subplots C, F, I and L display the histograms of the distribution function for log[A(*m*)_green _].

Wavelet coefficients A(*m*)_red _vs. A(*m*)_green _(*m *= 8,..., 11) for the signal on the entire array are plotted in Figure 3 (subplots A, D, G, J). Each point on the graph corresponds to the pair of the wavelet coefficients of the signals of the red and green channels on the tiling array. As can be seen from the graph, the majority of the points are located inside of the triangle-like area bounded by two lines coming from the origin of the coordinates. Plotting the same data using logarithmic coordinates log[A(*m*)_red _/A(*m*)_green _] vs. log[A(*m*)_green _], we see that all points lay inside of the stripe-shaped region and that the width of this region is roughly independent of the average intensity of the signal (see Figure 3 (subplots B, E, H and K)). Figure 3 (subplots C, F, I and L) shows the histograms of the distribution function for the log[A(*m*)_green _]. Peaks on the histograms for log[A(*m*)_green _] at the resolution levels *m *= 8,9,10 indicate that the scale of the wavelet at those resolution levels is smaller than the size of many contiguously tiled regions on the ENCODE array. Many data points inside the red box in Figure 3 (subplots B, E, H and K) correspond to the peaks inside the contiguously tiled regions whose size is larger than the size of the wavelet used for the signal decomposition. We can only identify parts of the broad peaks by going back to the original input signal and selecting the regions corresponding to those wavelet coefficients. In order to identify all the broad peaks in their entirety we should combine the information provided by the wavelet coefficients up to the resolution level *m *= 11. As can be seen on Figure 3 (subplot L) the histogram for A11 becomes flat compared to the histograms for A10, A9 and A8 (subplots C, F and I). This is an indication that there is a lack of contiguously tiled regions on the Encode tiling array with a size greater than the size of the wavelet at the resolution level *m *= 11. Wavelet coefficients for the decomposition levels m > 12 contain the information from the missing data regions where the signal is zero. Consequently, It makes it impractical to use the decomposition levels m > 12.

**Figure 4:**
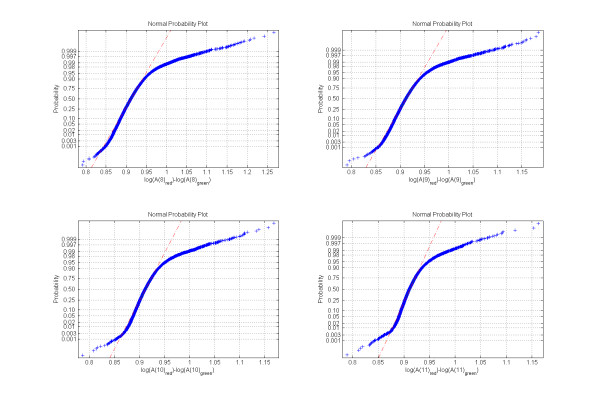
**Normality test for log[A(*m*)red/A(m)green], *m *= 8,.., 11**. If the data are normal the plot will be linear. The probability density function of log[A(*m*)_red _/A(*m*)_green _] is very close to normal distribution at each resolution level *m *= 8,..., 11. The deviation from the normal distribution is due to the regions of high enrichment attributable to the specific hybridization.

The probability density function of log[A(*m*)_red_/A(*m*)_green _] is very close to normal at the resolution levels *m *= 8,..., 11; as can be seen in Figure 4. The deviation from the normal distribution is due to the regions of high enrichment attributable to the specific hybridization. For each resolution level *m *we can compute the standard deviation *σ_m _*of log[A(*m*)_red _/A(*m*)_green _] and threshold the wavelet coefficients relative to *σ_m_*, allowing us to obtain regions of biochemical activity of interest at the same confidence level for all relevant length-scales. For every wavelet coefficient above the threshold we can go back to the original signal and identify the region of the biochemical activity. The size of each region is related to the resolution level of the corresponding wavelet. At the end, all the detected regions are combined together.

The log-normal distribution is the characteristic feature of multiplicative random processes [16]. One explanation for the appearance of the log-normal distribution in the data is that the measurement process of the fluorescent signal of the tiling array involves multiplicative random factors. These factors can include the collection efficiency of the light during array scanning and the variation of the quantum efficiency of the pixels in the CCD camera. The log-normal distribution was previously observed in the fluorescence microscopy signal [17].

Furthermore, the log-normal distribution of the data could be attributed to the kinetics of the hybridization process on the array.

### Consistency property of the wavelet coefficients corresponding to the locations of the peaks

**Figure 5:**
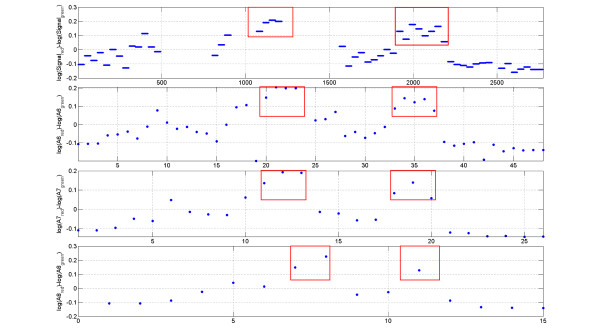
**Illustration of the consistency property for the wavelet coefficients corresponding to a region of biochemical activity**. We used the same genomic region as in Figure 2. The red box indicates the hit regions.

A very interesting feature can be observed from Figure 5: Approximation coefficients for the red channel are consistently above the approximation coefficients for the green channel over the region of the biochemical activity across several wavelet decomposition levels. We use this characteristic to decrease the number of false-positive calls. We describe the numerical procedure ensuring the consistency property of the wavelet coefficients below.

According to (2.1) the approximation coefficients *A*(*m*+1)*_n_*at scale (*m*+1) can be obtained from six approximation coefficients at the finer resolution scale (*m*):

$$A{\left(m+1\right)}_{n}=\frac{1}{\sqrt{2}}\sum_{k}c_{k}A{\left(m\right)}_{2n+k}$$

where *c_k_*, *k *= 1,.., 6 are the decomposition low-pass filter coefficients of Coif1: *c*_1 _= -0.0157, *c*_2 _= -0.0727, *c*_3 _= 0.3849, *c*_4 _= 0.8526, *c*_5 _= 0.3379, *c*_6 _= -0.0727.

Three of the six approximation coefficients at the resolution level m: *A*(*m*)_2*n*-1_, *A*(*m*)_2*n*-2 _and *A*(*m*)_2*n*-3 _contribute the most to *A*(*m*+1)*_n_*. Repeating the same argument for the decomposition level *m *we find nine approximation coefficients at the resolution level *m-1 *contributing mostly to the numerical values of three approximation coefficients at the resolution level *m*. For the wavelet decomposition using Coif1 there are nine approximation coefficients at the resolution level *m-1 *and three approximation coefficients at the resolution level *m *that greatly influence the numerical value of the approximation coefficient *A*(*m*+1)*_n_*.

### Description of the numerical algorithm

Our algorithm performs wavelet decomposition of the signals in red and green channels, computes the standard deviations of the distribution functions of the log-ratios of of the wavelet coefficients, thresholds the log-ratios, checks the thresholded wavelet coefficients for consistency, generates hit regions from the wavelet coefficients selected by the algorithm and estimates the FPR (false positive rate) for the chosen threshold.

Here are the steps of our algorithm:

1) Depending on the range of peaks we are looking for, we choose the range of wavelet decomposition levels: *M*_1 _≤ *m *≤ *M*_2_. The width of the wavelet at the decomposition level m is approximately *2^m^*. Hence, we use wavelet decomposition levels *M*_1 _≤ *m *≤ *M*_2 _to look for the peaks of width *L*: $2^{M_{1}}\leq L\leq 2^{M_{2}}$. For example, we use *M*_1 _= 8, *M*_2 _= 11 for POL II data and *M*_1 _= 8, *M*_2 _= 12 for the histone modification data. We compute the wavelet decomposition of the signal at levels *M*_1 _- 2 ≤ *m *≤ *M*_2 _(two extra levels *M*_1 _- 1 and *M*_1 _- 2 are computed to check the wavelet coefficients at level *M*_1 _for consistency).

2) For every decomposition level m, the probability density function of $\log\left(\frac{\left(A{\left(m\right)}_{n\;red}\right)}{\left(A{\left(m\right)}_{n\;green}\right)}\right)$ is approximated by a Gaussian and the standard deviation *σ_m _*is computed.

3) We call a region corresponding to the approximation coefficient *A*(*m*)*_n _*a hit only if:

a) $\log\left(\frac{\left(A{\left(m\right)}_{n\;red}\right)}{\left(A{\left(m\right)}_{n\;green}\right)}\right)>\alpha \sigma_{m}$, where *α *is the numerical value of the threshold. The threshold value is the same for all *M*_1 _- 2 ≤ *m *≤ *M*_2_. The thresholding allows us to select peaks of different sizes with the same confidence level.

b) The same as in 1) is true for the log ratios of at least three approximation coefficients at the resolution levels *m-1 *and *m-2 *contributing greatly to *A*(*m*)*_n_*. Requirements a) and b) impose consistency constraints on the wavelet coefficients across three resolution levels which help to reduce the number of false positives. Each time we find a hit we go from *A*(*m*)*_n_*, to *A*(*m*-1)_2*n*-1_, *A*(*m*-1)_2*n*-2_, and *A*(*m*-1)_2*n*-3_; until we reach the original input signal to identify the region of biochemical activity.

4) We combine together each overlapping group of hits into one big hit region. We call N the total number of final hit regions.

We can estimate the false positive rate (FPR) corresponding to the chosen value of threshold *α*. Signal of the red channel is randomly shuffled between the probes. We repeat the same scoring procedure for the shuffled signal keeping *M*_1_, *M*_2 _and *α *the same.

The obtained hits are false hits which allow us to estimate the false positive rate (FPR): $FPR=\frac{N_{shuff}}{N}$, where *N *is the total number of hits without random shuffling and *N_shuff _*is the total number of hits after the random shuffling of the red signal probes. One can choose the *α *according to the corresponding FPR.

Figure 5 shows Pol II binding sites located between genomic coordinates 1900 and 2200 and also between 1050 and 1250. Two regions are identified as hits by our algorithm with parameters *M*_1 _= 8, *M*_2 _= 11 and the threshold *α *corresponding to the estimated FPR = 5%. The wavelet coefficient which satisfy conditions 3) and the regions of the signal corresponding to those wavelet coefficients are indicated by the red boxes.

**Figure 6:**
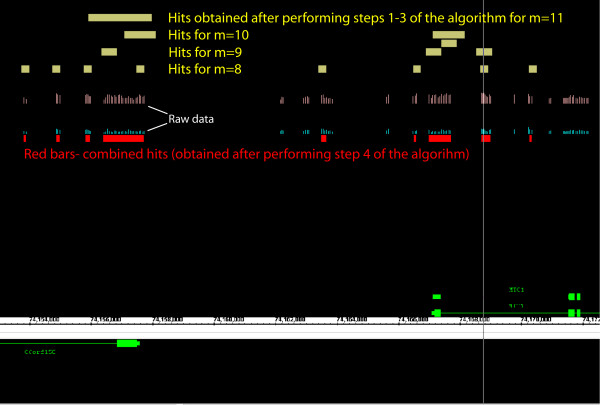
**IGB snapshot of the signal on tiling array and hit regions**. Raw signals for the green and red channels are shown as blue and pink tracks. Yellow bars indicate the hit regions obtained using wavelet decomposition at the resolution levels *m *= 8, ..., 11. Hit regions obtained by combining the information from these resolution levels are shown as red bars.

Figure 6 shows a snapshot from the Affymetrix Integrated Genome Browser (IGB) displaying broader peaks of the Pol II ChIP-chip data using the Nimblegen ENCODE tiling array. Raw signals for the green and red channels are shown as blue and pink tracks. We used again the following set of parameters: *M*_1 _= 8, *M*_2 _= 11 and the threshold *α *corresponding to the estimated FPR = 5%. Yellow bars indicate the hit regions corresponding to the wavelet coefficients at the resolution levels *m *= 8, ..., 11 satisfying the condition 3) of our numerical algorithm. Hit regions obtained by combining the information from these resolution levels (combining together overlapping yellow bars) are shown as red bars (step 4 of our algorithm).

### Comparison with other methods

In order to test the performance of our method with the consistency constraint previously described, we applied our algorithm to the Spike-in data from the ENCODE Nimblegen tiling array. Mixtures of human genomic DNA and 100 human sequences at various concentrations were hybridized on the array [5]. Spike-in data was obtained from human sequences of approximately the same size, which were generated in the laboratory. Spike-in data allows for an objective estimation of the performance of our method and a comparison with other methods.

We used model parameters: *M*_1 _= 8, *M*_2 _= 9 (to identify narrow peaks) and varying *α *to obtain ROC-type curve. We choice of the model parameters was based on our observation that the size of the wavelets should be comparable to the size of the peaks to identify. ROC-type curves were generated by plotting the Sensitivity (i.e. the number of true positives/100) vs. the False Positives ratio (i.e. the number of false positives/100). The optimal ROC-type curve is the one closest to the left upper corner.

We compared our method with other methods described in [5]: MA2C, Splitter, Permu, ACME, TAMALg, and TAMALs. MA2C (Model-based Analysis for 2-Color arrays) [8] is a normalization method based on the GC content of the probes. It compensates each probe's log(Cy5/Cy3) ratio for the GC bias and weights each probe, taking into account the signal variance of the GC group to which the probe belongs. A sliding window consisting of 500 bp was used, and windows with high median values were identified as hits. The Splitter algorithm [7] incrementally changes the cutoff value of the signal and compares the total number of hits before and after the change. If the ratio of the number of hits before and after the cutoff change is smaller than a pre-defined value, the algorithm stops and hits before the last cutoff change are reported as final hits. Clusters of probes located closer than a "maxgap" parameter were merged together. Clusters of probes containing the number of probes smaller than a "minrun" parameter were discarded. Permu [6] identifies the peaks within the sliding window based on iterative thresholding procedure. FPR (false positive rate) is assigned to each peak using the randomized data. TAMALg and TAMALc are two versions of the same peak-finding algorithm [18] that use different stringency levels.

**Figure 7:**
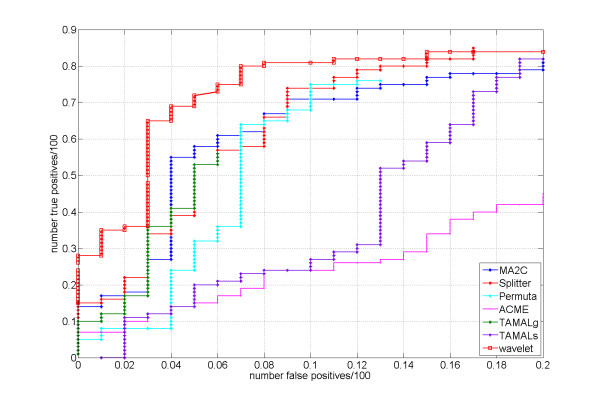
**Roc-like curves generated from the Spike-in data experiments data**. Our method demonstrates excellent performance compared to other methods, as shown by the ROC-type curves.

Our method demonstrated excellent performance compared to other methods as can be seen from the ROC-type curve in Figure 7. The intuitive reason behind of such a good performance of our method is that the shape of the wavelets we use is very similar to the shape of peaks of the signal. Another reason of a good performance is the use of consistency constraint which reduces the number of false positives.

We also tested our method using STAT1 data from ENCODE Nimblegen arrays in [19]. We use the following parameters for our model: *M*_1 _= 8, *M*_2 _= 12 and the threshold *α *corresponding to the estimated false positive rate FPR = 5%. Most of the hits (84%) obtained by our method overlap with hits reported in [19].

## Conclusions

We analyzed tiling array data using wavelet transformations, and from the resulting wavelet coefficients we obtained clear intensity and length-scale separation between the background signal and the signal coming from the regions of biochemical activity. A thresholding procedure was applied to the wavelet coefficients at different resolution levels with the consistency constraint in order to delineate the regions of biochemical activity of interest at the same confidence level for all the relevant length-scales. This method was successfully applied to several ChIP-chip data sets, including Pol II and histone modification experiments. Our method demonstrated excellent performance using Spike-in data from the Nimblegen tiling array.

## Authors' contributions

AK designed the algorithm, coded it and wrote the manuscript. JR provided crucial help in improving the data analysis. MG provided general advice and guidance in drawing necessary conclusions and results. All the authors read and approved the final manuscript.

## Supplementary Material

Additional file 1 **Figure S1 - High-pass and low-pass decomposition and reconstruction filters for Coif1**. Low-pass decomposition filter *c *has a triangular-like shape that resembles the shape of the signal over binding sites observed in ChIP-chip experiments. Click here for file
