# Supplementary figures and images for: Tiling array data analysis: a multiscale approach using wavelets
Source: BMC Bioinformatics. 2011 Feb 21;12:57. doi: 10.1186/1471-2105-12-57 (PMC3055839; doi:10.1186/1471-2105-12-57)

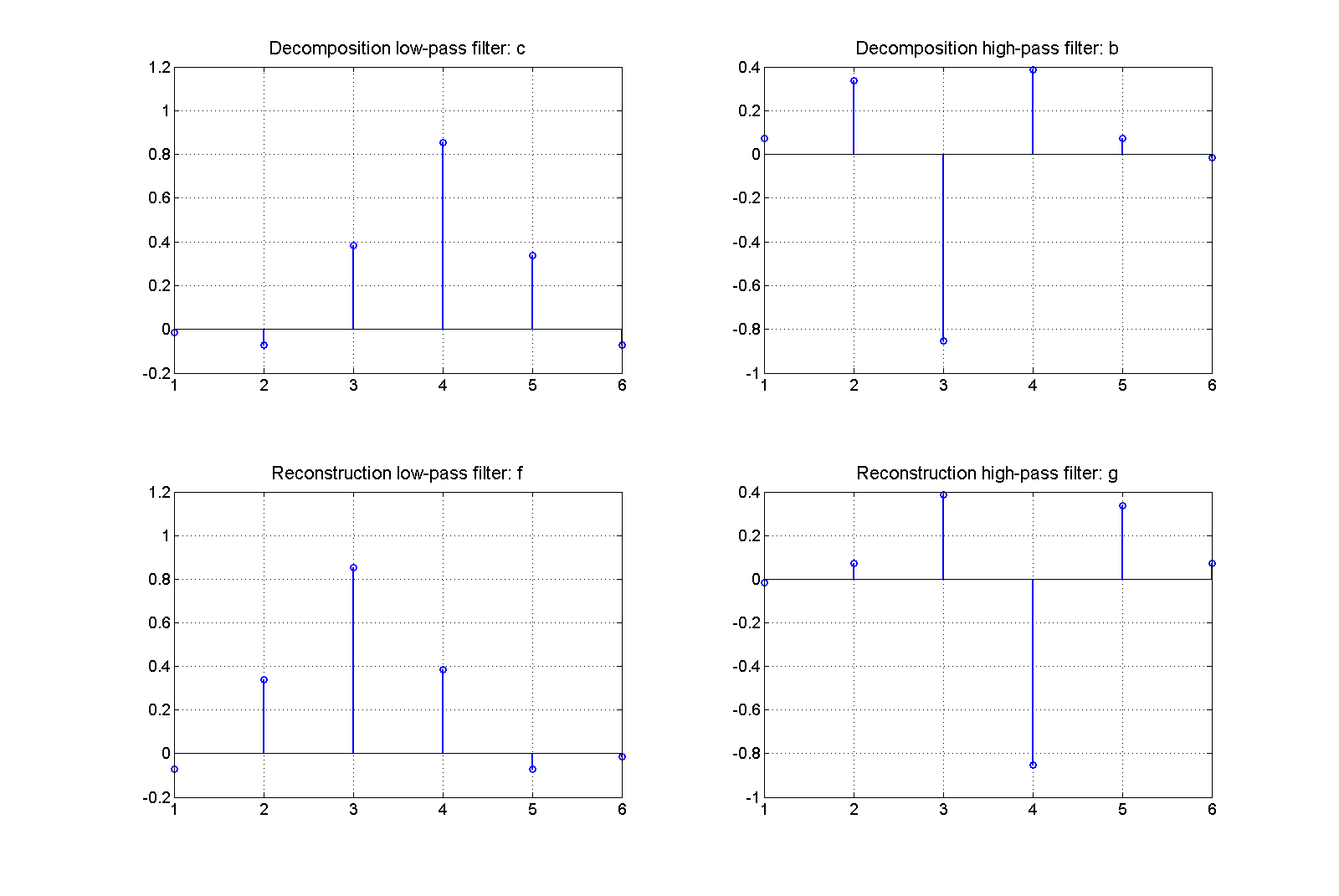

Supplement: Additional file 1 — Figure S1 - High-pass and low-pass decomposition and reconstruction filters for Coif1. Low-pass decomposition filter c has a triangular-like shape that resembles the shape of the signal over binding sites observed in ChIP-chip experiments. [file 1471-2105-12-57-S1.TIFF]
